# Infectious Diseases, Preterm Delivery, and Infant Outcomes^1^

**DOI:** 10.3201/eid1011.040624_10

**Published:** 2004-11

**Authors:** David Eschenbach, Michael Gravett, Rodney Willoughby

**Affiliations:** * University of Washington, Seattle, Washington, USA;; † Oregon Health Sciences University, Portland, Oregon, USA;; ‡ Johns Hopkins Hospital, Baltimore, Maryland, USA

**Keywords:** infectious diseases, preterm delivery, infant outcomes

## Infectious Diseases and Preterm Delivery in Industrialized and Developing Countries

Preterm delivery remains the most important single issue in perinatal care because 70% of all perinatal deaths and a similar proportion of perinatal illness occur in the preterm delivered infant. Infants born very prematurely at <30 weeks' gestation or with a very low birth weight of <1,500 g have the highest rates of perinatal illness and death.

## Chorioamnion Infection in Premature Birth and Disease in Newborns

The association between infection in the lower genital tract, in the chorioamnion of the placenta or in amniotic fluid, and preterm birth is both strong and consistent. Chorioamnion or amniotic fluid infection, or both, is especially common in infants of very low birth weight: 30%–50% of infants born at <1,500 g have chorioamnion or amniotic fluid infection. However, a causal relationship between infection and preterm birth is not established. Antibiotic treatment trials generally have not reduced preterm delivery because this relationship is complex, as individual responses occur to infections vary widely, and host response is a key, understudied factor.

## Models for Intervention

A primate experimental model of infection and preterm delivery indicates that bacteria experimentally placed into amniotic fluid elicit an almost immediate cytokine response, a fetal cortisol response, little or no maternal effects of infection, and a delay in uterine contractions for 30–40 hours, after which labor ensues. Such models are important to detect early proteins in infection, to understand the mechanisms by which infection causes preterm birth and the fetal consequences of intrauterine infection. For example, amniotic fluid infection induces interleukin-1, prostaglandin, uterine contractions, and preterm delivery. Interleukin (IL)-1β infusion alone without infection causes the release of cytokines and prostaglandins into amniotic fluid with uterine contractions, but it does not cause preterm delivery. Dexamethasone and interleukin-10 infused into amniotic fluid together with IL-1β inhibit intrauterine IL-1β and prostaglandins and reduce uterine contractions. Thus, dexamethasone and IL-10 infusion could help inhibit infection-induced preterm labor if amniotic fluid infection were controlled.

## Infectious Diseases and Cerebral Palsy in Industrialized and Developing Countries

Fetal infection in utero has recently been associated with several severe neonatal effects. Intrauterine infection is associated with respiratory distress, necrotizing enterocolitis, and neonatal encephalopathy. Chorioamnionitis has been associated with cerebral palsy, a serious permanent neurologic condition also associated with preterm birth. In term infants, clinical chorioamnionitis is highly associated with cerebral palsy. In preterm infants, clinical chorioamnionitis is less strongly and consistently associated with cerebral palsy. These findings indicate that infection has a major effect on the infant besides the already large issue of prematurity.

The incidence of infection during pregnancy and its impact on preterm delivery and neonatal effects in developing countries is largely unknown. However, the prevalence of lower genital infection is more common in developing than in industrialized countries. In addition, other systemic infections are more common in developing than in industrialized countries during pregnancy, for example, malaria, tuberculosis, HIV, and diarrheal infectious disease.

Thus, in developing countries, infection in pregnancy may have a large impact on the neonate, not only by causing intrauterine infection and preterm delivery, but also by causing direct placental infection in the case of malaria. In developing countries, infection also is more likely to cause serious maternal disease, which, in turn, leads to preterm delivery or to serious fetal infection.

